# Dry habitats were crucibles of domestication in the evolution of agriculture in ants

**DOI:** 10.1098/rspb.2017.0095

**Published:** 2017-04-12

**Authors:** Michael G. Branstetter, Ana Ješovnik, Jeffrey Sosa-Calvo, Michael W. Lloyd, Brant C. Faircloth, Seán G. Brady, Ted R. Schultz

**Affiliations:** 1 Department of Biology, University of Utah, Salt Lake City, UT 84112, USA; 2 Department of Entomology, National Museum of Natural History, Smithsonian Institution, Washington, DC 20560, USA; 3 Department of Entomology, University of Maryland, College Park, MD 20742, USA; 4 Center for Social Insect Research, School of Life Sciences, Arizona State University, Tempe, AZ 85287, USA; 5 Department of Biological Sciences and Museum of Natural Science, Louisiana State University, Baton Rouge, LA 70803, USA

**Keywords:** attine ants, fungus farming, phylogenomics, ultraconserved elements, symbiosis

## Abstract

The evolution of ant agriculture, as practised by the fungus-farming ‘attine’ ants, is thought to have arisen in the wet rainforests of South America about 55–65 Ma. Most subsequent attine agricultural evolution, including the domestication event that produced the ancestor of higher attine cultivars, is likewise hypothesized to have occurred in South American rainforests. The ‘out-of-the-rainforest’ hypothesis, while generally accepted, has never been tested in a phylogenetic context. It also presents a problem for explaining how fungal domestication might have occurred, given that isolation from free-living populations is required. Here, we use phylogenomic data from ultra-conserved element (UCE) loci to reconstruct the evolutionary history of fungus-farming ants, reduce topological uncertainty, and identify the closest non-fungus-growing ant relative. Using the phylogeny we infer the history of attine agricultural systems, habitat preference and biogeography. Our results show that the out-of-the-rainforest hypothesis is correct with regard to the origin of attine ant agriculture; however, contrary to expectation, we find that the transition from lower to higher agriculture is very likely to have occurred in a seasonally dry habitat, inhospitable to the growth of free-living populations of attine fungal cultivars. We suggest that dry habitats favoured the isolation of attine cultivars over the evolutionary time spans necessary for domestication to occur.

## 1. Introduction

During the past 10 000 years, humans have domesticated over 260 plant, 470 animal and 100 mushroom-forming fungal species [1–3]. Humans have modified these domesticates through diverse conscious or unconscious programmes of artificial selection that required, at least temporarily, reduced gene flow between populations of domesticates and those of their free-living progenitors [4]. Barriers to gene flow have included the isolation of domesticates in discrete garden plots and livestock pens accompanied by programmes of selective breeding, in some cases via asexual propagation, self-fertilization, and the propagation of reproductively isolated polyploid and translocation races. In a subset of cases, barriers to gene flow have also included the separation of domesticates from their free-living conspecifics by allopatry (i.e. by the transport, whether deliberate or incidental, of the domesticates to localities at the peripheries of or completely outside of their ancestral ranges) [5–9]. With the advent of genomics, historical patterns of domestication by humans are the focus of reinvigorated research [10–13]. Here, we examine phylogenetic patterns of non-human domestication of fungi by ants that may provide insights into the dynamics underlying similar processes of domestication in other animal groups such as termites, bark beetles and even bees [14–18].

Fungus-farming ‘attine’ ants (Formicidae: Myrmicinae: Attini: Attina) are a monophyletic subtribe of approximately 250 described New World species that cultivate fungi for food [19]. Although the ants are obligate symbionts, their fungal cultivars (Agaricaceae: Leucocoprineae and Pterulaceae: *Pterula*) vary in symbiotic commitment in a pattern that is highly correlated with ant and fungal phylogenies [20–23]. The multiple species of fungi associated with the most primitive fungus-farming ants are, so far as is known, facultative symbionts (i.e. capable of a free-living existence outside of the symbiosis). Because these ‘lower’ attine fungi have been shown to be freely interbreeding members of larger, conspecific, free-living populations, they are usually regarded as non-domesticated [19,24,25]. In contrast, the clade of fungal cultivar species associated with so-called ‘higher’ attine ants, including the well-known leaf-cutting ant genera *Atta* and *Acromyrmex*, have become polyploid, obligate symbionts, and are no longer capable of living apart from their ant hosts. Higher attine fungi represent the best-confirmed case of domestication in attine ant agriculture and they have become the subjects of intensive study including proteomic and genomic investigation [26–29].

Ant agriculture is hypothesized to have arisen in the wet forests of tropical South America approximately 55–65 million years ago [16,23,27,30–33]. Most subsequent attine agricultural evolution, including the domestication event that produced the ancestor of the higher attine cultivars, is likewise hypothesized to have occurred in South American rainforests because ant-cultivated fungi are thought to be native to such habitats and incapable of surviving elsewhere as free-living organisms [30,34–37]. This ‘out-of-the-rainforest’ hypothesis is consistent with the observation that of all South American habitats (including deserts and seasonally dry habitats), wet Neotropical forests are home to the highest diversity and abundance of species in the fungal tribe Leucocoprineae, from which the ancestral attine cultivars arose [20,24,38–41]. The hypothesis is also consistent with the fact that all known free-living close relatives of attine fungal cultivars, including conspecifics of ant-cultivated fungi, have been collected in the wet forests of Panama and Brazil [24]. The out-of-the-rainforest hypothesis presents a problem, however, for explaining the domestication event that resulted in the origin of higher attine fungi and for ant–fungus coevolution, more generally. If domestication requires reproductive isolation of the domesticate from its free-living progenitor, and if, in wet forests, an ant-cultivated fungal species is genetically connected to a larger population of a free-living fungal species, how could domestication have occurred?

Here, we describe the results of a phylogenomic analysis of the fungus-farming ants, in which we used hundreds of ultra-conserved element (UCE) loci to resolve outstanding ambiguities in attine ant phylogeny. This robust phylogeny permits the identification of major evolutionary transitions on specific lineages, including the transition from lower to higher agriculture, with greatly reduced phylogenetic uncertainty. This phylogeny also allows the reconstruction of the ancestral habitats and areas in which these transitions is likely to have occurred. We find that the out-of-the-rainforest hypothesis is supported with regard to the origin of attine ant agriculture; however, contrary to expectation, we find that the transition from lower to higher agriculture is very likely to have occurred in a seasonally dry habitat (cerrado or savannah), inhospitable, at least during the dry season, to the growth of free-living populations of attine fungal cultivars. We suggest that inhospitable habitats favoured the isolation of attine cultivars over the evolutionary time spans necessary for domestication to occur. We also identify the sister group of attine ants with high confidence, providing a new target for investigations into the biological traits that promoted fungus farming.

## 2. Material and methods

### (a) Taxon sampling

We chose a total of 119 taxa for inclusion in our study, representing a broad range of fungus-farming and non-fungus-farming ant species (electronic supplementary material, table S1). Within the fungus-farming ants, we included 78 species, covering all 16 genera and including the morphologically enigmatic taxa *Mycetosoritis asper*, *M. explicatus* and *Paramycetophylax bruchi*. For *Cyphomyrmex* and *Trachymyrmex*, two genera that are known to be non-monophyletic, we sampled broadly across all clades identified in a previous study [23]. Outside of the fungus-farming ants, we included 41 outgroup species, 29 from within the tribe Attini and 12 from outside of this group. We chose genus-level outgroups from within the Attini to match the extensive sampling of Ward *et al*. [42], missing only the genera *Diaphoromyrma*, *Mesostruma* and *Talaridris*.

### (b) Library preparation, UCE enrichment and sequencing

We employed the UCE approach to phylogenomics [43,44], combining target enrichment of ultra-conserved elements (UCEs) with multiplexed, next-generation sequencing. For UCE enrichment, we used an RNA bait library for Hymenoptera that targets 1510 UCE loci [44]. The laboratory protocol we used closely follows the methods reported in [44], and we provide a detailed description of the protocol in electronic supplementary material, appendix A2.

### (c) Bioinformatics and matrix preparation

The sequencing centres demultiplexed and converted raw Illumina data from BCL to FASTQ format. Starting with the FASTQ files, we performed all initial bioinformatics steps using the Phyluce v. 1.4 software package [45] and associated programs (see electronic supplementary material, table S2 for all sequencing and assembly statistics). We cleaned and trimmed raw reads using Illumiprocessor [46] and assembled contigs *de novo* using Trinity v. r2013-02-25 [47]. After assembly, we used several Phyluce scripts to identify and extract UCE contigs, remove potential paralogs, and add in data from two genome-enabled taxa (*Atta cephalotes* and *Acromyrmex echinatior*; see electronic supplementary material, table S3). We aligned the UCE loci using MAFFT v. 7.130b [48] and trimmed the alignments with Gblocks v. 0.91b [49,50] using reduced stringency settings. We filtered the master set of alignments for varying levels of taxon occupancy (percentage taxa required to be present in a given locus) and selected the 75% filtered alignment set as the main set for phylogenetic analysis (‘Attine-118T-F75’; see electronic supplementary material, table S4 for all matrix statistics). The Attine-118T-F75 alignment set includes 950 loci and 652 774 bp of sequence data, of which 305 858 sites are informative. See electronic supplementary material, appendix A2 for additional detail on matrix preparation and taxon occupancy filtering.

### (d) Phylogenetic analyses

Using the Attine-118T-F75 alignment set, we investigated the effects of inference method and data partitioning on results (see electronic supplementary material, appendix A2 for additional detail). Note that we excluded one taxon from this focal alignment set (*Paramycetophylax bruchi*) because it received few captured UCE loci (electronic supplementary material, table S2). We did, however, include this taxon in a separate, 119-taxon analysis, described below.

For phylogeny estimation, we compared maximum likelihood (ML), Bayesian inference (BI) and species tree (ST) approaches. For concatenated ML analyses, we compared four partitioning schemes using RAxML v. 8 [51]: unpartitioned, partitioned by locus, partitioned by PartitionFinder v. 2.0 using the rcluster algorithm (PF; data pre-partitioned by locus) [52,53], and partitioned by PF v. 2.0 using the kmeans algorithm [54]. For each analysis, we executed a rapid bootstrap plus best tree search (‘-f a’ option), and we used the GTR + *Γ* model of sequence evolution (for both best tree and bootstrap searches). To address the concern that bootstrap scores can be misleading with phylogenomic data [55], we also performed a jackknifing analysis, in which we randomly sampled subsets of loci multiple times (100 replicates of 100 UCE loci). For BI analyses we used ExaBayes v. 1.4.1 [56], and performed unpartitioned and partitioned searches on the concatenated matrix. For the partitioned searches we used the same kmeans-partitioning scheme that we used with RAxML. We selected kmeans because the tree resulting from the kmeans-partitioned ML analysis had the highest likelihood score and reasonable branch length estimates (electronic supplementary material, table S5). For the partitioned BI searches, we performed two analyses, one with parsimony starting trees and one with random starting trees. We assessed run performance by examining log files with Tracer v. 1.6.0 [57]. We performed ST analyses using the program ASTRAL v. 4.8.0 [58,59]. First, we generated gene trees using RAxML and then we used only the 500 gene trees with the highest mean bootstrap scores (calculated in R v. 3.2.2 [60] using a script modified from [61]) as input into ASTRAL. We included this step to reduce noise introduced by including uninformative loci (see [62]), and we conducted the ASTRAL analysis with 200 multi-locus bootstrap replicates [63].

To explore our data for other potential biases, we generated two additional matrices. First, we used the program BaCoCa [64] to identify any loci exhibiting significant deviations from base composition heterogeneity (*χ*^2^ test, *p* < 0.05). After removing biased loci (36 total), we concatenated the remaining 914 loci for analysis (‘Attine-118T-F75-975’). Second, to control for either base composition heterogeneity or saturation, we converted the concatenated Attine-118T-F75 matrix to RY-coding (‘Attine-118T-F75-RY’). We analysed both of the above matrices unpartitioned with RAxML.

To place *Paramycetophylax bruchi* (excluded due to poor UCE capture) in the attine tree we performed one additional analysis with this taxon included. We generated a new set of alignments filtered at 75% taxon occupancy (‘Attine-119T-F75’), concatenated the loci, and then performed an unpartitioned analysis with RAxML.

### (e) Divergence dating

We generated a time tree for the evolution of fungus-farming ants using BEAST v. 1.8.2 [65]. To calibrate the analysis, we used nine fossil calibrations, and one secondary calibration (electronic supplementary material, table S6). To decrease computation time we (i) used a constraint tree and turned off tree search operators, and (ii) used subsets of UCE loci rather than the entire matrix. For five separate matrices (each with 20 randomly selected UCE loci), we performed two independent BEAST runs, each progressing for 50 million generations. We assessed burn-in, convergence among runs, and run performance by examining log files with Tracer v. 1.6. We generated chronograms for each of the five matrices separately and for all runs combined. For additional detail, see electronic supplementary material, appendix A2.

### (f) Historical biogeography

We inferred the biogeographic history of the fungus-farming ants using the R package BioGeoBEARS (BGB) [66,67]. For the tree, we used the BEAST time tree pruned to include only the fungus-farming ants and their sister group. We coded taxa for the following areas: (A) Nearctic; (B) Middle America (including the Caribbean); (C) South America; (D) Afrotropics; and (E) Australasia. Using BGB, we compared six different biogeographic models: DEC [68], DEC + J, DIVALIKE [69], DIVALIKE + J, BAYAREA [70] and BAYAREALIKE + J. For each model we performed a time-stratified analysis using the time periods 0–5 Ma, 5–35 Ma and 35–65 Ma, which correspond to post-closure of the Isthmus of Panama [71], pre-closure of the Isthmus of Panama and pre-glaciation of Antarctica [72], respectively. For additional detail, see electronic supplementary material, appendix A2.

### (g) Ancestral state reconstruction

We performed trait reconstruction analyses to examine the evolution of attine ant (i) agriculture and (ii) habitat preference. In both cases, we used the same pruned BEAST tree that we used for biogeography. For agriculture, we coded taxa as practising (0) no agriculture, (1) lower agriculture, (2) coral-fungus agriculture, (3) yeast agriculture, (4) higher agriculture or (5) leaf-cutter agriculture (agricultural systems reviewed in [23]). We then used the ‘ace’ function from the R package APE [73] to test three different reconstruction models: equal rates (ER), symmetrical rates (SYM) and all rates different (ARD). For habitat preference, we coded taxa as occurring in: (A) continuously wet habitat (rainforest), which should be hospitable all year long to free-living populations of attine ant fungal cultivars; (B) seasonally dry habitat (e.g. cerrado, savannah, desert, dry scrub), which would be inhospitable, at least for part of the year, to free-living cultivars; or (AB) both wet and dry habitats. We inferred ancestral habitat preference using BGB, because it more realistically treats habitat AB as a combination of habitats rather than as a distinct, third character state. We performed six analyses comparing the same models used for biogeographic inference.

### (h) Diversification rates

We investigated diversification dynamics in the fungus-farming ants using two approaches. We tested for significant shifts in diversification rates across the entire tree using the R package TreePar [74], which is an ML-based program that allows for non-constant diversification rates and incomplete taxon sampling. We also tested for rate shifts among lineages using the Bayesian program BAMM v. 2.5 [75–78] (see also [79,80]) and the accompanying R package BAMMtools [81]. A useful feature of BAMM is that it allows for non-random, incomplete taxon sampling via the input of a sampling probability file, which we incorporated into our analysis (electronic supplementary material, table S7). For additional detail, see electronic supplementary material, appendix A2.

## 3. Results

### (a) Phylogeny of the fungus-farming ants

**Figure 1.**
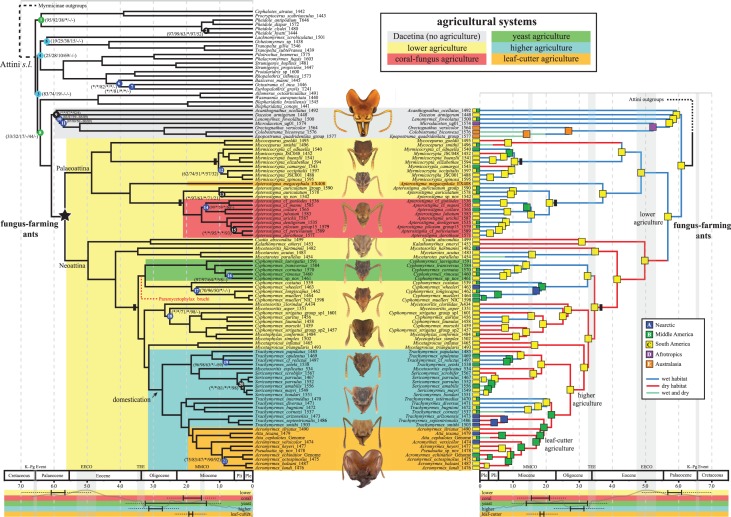
Evolution and biogeography of the fungus-farming ants and their agricultural systems. The tree topology (left and right sides) matches the best tree topology recovered from all partitioned RAxML analyses of the Attine-118T-F75 matrix (950 UCE loci, 652 774 bp). We estimated divergence dates using BEAST and 10 node calibrations (electronic supplementary material, table S6). *Left side*: numbered nodes received less than 95% support in at least one of six analyses (raxml-rcluster/raxml-kmeans/raxml-jacknife/exabayes-kmeans/raxml-ry-coding/astral) and the colour of the node corresponds to the frequency with which that node was recovered across 10 analyses (electronic supplementary material, table S8; black = 10/10, purple = 8–9/10, green = 5–7/10, sky blue = < 5/10). The asterisk (*) signifies 100% clade support and the dash (-) signifies that the clade was not recovered in the best tree. We mapped the five distinct attine ant agricultural systems (lower agriculture, coral-fungus agriculture, yeast agriculture, higher agriculture and leaf-cutter agriculture) onto the tree using ML-based trait reconstructions. *Right side:* coloured squares indicate current or ancestral geographical ranges, with the ancestral ranges inferred using the program BioGeoBEARS (DEC + J model). We used the following ranges: (A, blue) Nearctic, (B, green) Middle America, (C, yellow) South America, (D, purple) Afrotropics and (E, orange) Australasia. Coloured branches indicate current and ancestral habitat preference (blue, wet habitat; red, dry habitat; turquoise, wet and dry habitats), with ancestral preference inferred using BioGeoBEARS (BAYAREALIKE + J model). *Both sides:* The bars at the bottom of each chronogram provide stem- and crown-group ages for each agricultural system. Dotted lines correspond to the 95% HPD of the BEAST divergence date estimates. The wavy, light grey line depicts average global temperature (adapted from [72]). For reference, major global events are highlighted on the geological timeline (EECO, Early Eocene Climatic Optimum; TEE, Terminal Eocene Event; MMCO, Mid-Miocene Climatic Optimum). The three vertical black bars on nodes mark rate shifts identified by BAMM (all rate increases).

All phylogenetic analyses (ML, BI and ST) recovered the fungus-farming ants as a highly supported clade, with the dacetines (referred to here as the subtribe Dacetina and including the genera *Acanthognathus*, *Colobostruma, Daceton*, *Epopostruma, Lenomyrmex, Mesostruma, Microdaceton*) as the sister group (figure 1; electronic supplementary material, figures S1–S11 and table S8), confirming a previous result [42]. Support for the monophyly of Dacetina was high (more than 90%) in all analyses except for the ST analysis (6%). Within the fungus-farming ant clade, there was no topological variability among analyses and nearly all nodes received maximum support. Most relationships are congruent with previous molecular studies [23,32,82], but we recovered a number of novel results within the neoattines. Most importantly, we confidently resolved the positions of major *Cyphomyrmex* lineages and several enigmatic species of *Mycetosoritis* for the first time (both genera are non-monophyletic). Relationships among remaining genera within the more inclusive tribe Attini were less well resolved. The tribe and several groups of genera within the tribe were recovered with high support, but most relationships among genus groups were poorly supported. We did not recover the sister group to the Dacetina + Attina clade with high confidence, although most ML analyses suggested that the *Blepharidatta* group (*Blepharidatta, Wasmannia, Allomerus*) was sister to Dacetina + Attina.

### (b) Divergence dating of major transitions in ant agriculture

We recovered roughly identical divergence dates from the five different data subsets that we analysed and from the combined averaging of all results (electronic supplementary material, table S9). Additionally, our ancestral reconstructions of ant agriculture returned essentially identical results across the three different models that we tested, with the ER model being statistically favoured (electronic supplementary material, table S10 and figures S12–S14). Our date and trait inferences are largely congruent with previous studies [23,32], except that we recovered slightly older dates for the origin of higher fungus farmers.

The Attini *sensu lato* evolved 66 Ma (56–76 Ma HPD) at the K–Pg boundary followed by what was a rapid radiation of all of the major genus groups and subtribes. The fungus-farming ants originated as lower fungus farmers at the end of the Palaeocene sometime between 61 Ma (52–70 Ma) and 57 Ma (48–66 Ma), and the two major clades within fungus-farming ants, the ‘palaeoattines’ and ‘neoattines’, evolved as lower fungus-farmers more or less simultaneously at 49 Ma (39–58 Ma) and 50 Ma (42–59 Ma), respectively. The transition from lower agriculture to pterulaceous (coral-mushroom) agriculture (*Apterostigma pilosum* group) occurred sometime between 21 Ma (17–26 Ma) and 16 Ma (11–20 Ma). Within the neoattines, the transition from lower agriculture to yeast agriculture (*Cyphomyrmex rimosus* group) occurred between 33 Ma (27–39 Ma) and 14 Ma (9–19 Ma). The evolution of higher agriculture occurred between 31 Ma (26–37 Ma) and 27 Ma (22–33 Ma) and the leaf-cutting ants (although not necessarily leaf-cutting agriculture [22,27,83]) evolved most recently during the Miocene between 19 Ma (15–24 Ma) and 18 Ma (14–22 Ma).

### (c) The biogeographic context of fungus-farming evolution

BioGeoBEARS model comparison favoured the DEC + J model as the best-fitting model for the biogeographic data (figure 1; electronic supplementary material, figures S17, S18 and table S10), and most models inferred similar scenarios of ancestral range evolution (electronic supplementary material, figures S15–S26). The fungus-farming ants originated in South America and have maintained a strong presence in the region, as evidenced by the fact that most clades also have South American origins (59 out of 80 nodes). The ancestors of both yeast-farming attines and coral-fungus-farming attines evolved in South America. Several species and clades, however, dispersed from South America into Middle America and less often into the Nearctic. One major clade inferred to have had an origin in Middle America is the *Cyphomyrmex wheeleri* group, which includes one species that occurs far into North America (*C. wheeleri*). Notably, the other major clade to originate in Middle America is nested inside of the higher fungus farmers and includes the leaf-cutter ants. This clade is composed of the *Trachymyrmex intermedius* group, the *T. septentrionalis* group, which is an exclusively Nearctic lineage, and the leaf-cutting ant genera *Atta* and *Acromyrmex*. The leaf-cutter ants also originated within Middle America with later dispersal into both South America (multiple times) and the Nearctic (at least twice).

For habitat preference, BGB model comparison favoured the BAYAREALIKE + J model, which produced results nearly identical to the next-most-favoured model, DEC + J (figure 1; electronic supplementary material, figures S27–S38 and table S10). The ancestral fungus-farming ants evolved in continuously wet rainforest habitat; however, the two major clades of fungus farmers, the palaeoattines and neoattines, show very different trends, with the palaeoattines diversifying predominately in wet habitat, and the neoattines shifting into and diversifying predominately in dry habitat. Within the neoattines, there were two radiations that occurred predominately in wet habitat, one in the *Cyphomyrmex rimosus* plus *C. wheeleri* groups and one in the *Trachymyrmex intermedius* group. In addition to the general trend of neoattines diversifying in dry habitat, we found that both higher agriculture and leaf-cutter agriculture originated in dry habitat, contradicting the prevailing view that most evolution and domestication in the fungus-farming ant–fungus mutualism occurred in South American rainforest.

### (d) Diversification dynamics of fungus-farming ants

The tree-wide diversification rate analysis using TreePar found one significant rate shift across the fungus-farming tree at 6.5 Ma (*p* < 0.01, electronic supplementary material, table S11), with the net diversification rate showing a slight decrease. The among-lineages diversification rate analysis using BAMM found evidence for three rate increases (figure 1; electronic supplementary material, figures S39–S43), with the three-shift model receiving the highest posterior probability when compared with competing models (electronic supplementary material, table S12). The rate shifts occurred along the branches leading to *Myrmicocrypta*, *Apterostigma* (minus *A. megacephala*), and the neoattines minus several depauperate lineages (*Cyatta*, *Kalathomyrmex*, *Mycetarotes*). Counter to our expectations, none of these shifts directly corresponded with major shifts in ant agriculture; however, the neoattine shift is loosely correlated with climate change (see discussion below).

## 4. Discussion

Our model-based results (figure 1) support the long-held (but previously untested) hypothesis that fungus farming originated in the rainforests of South America [30,34–37,84,85]. However, in stark contrast to the consensus view that most subsequent evolution also occurred in South American rainforests (although see [86]), we found that the ancestors of most major attine lineages, including the ancestors of higher fungus farmers and leaf-cutter ants, probably evolved in dry or seasonally dry habitat (cerrado, savannah, desert etc.). This result is significant because it suggests a possible mechanism for the origin of the symbiotically obligate, highly derived, higher attine fungi. As in human-mediated domestication, the domestication of fungi by ants almost certainly required, at least temporarily, the genetic isolation of fungal cultivars from their free-living, wild-type conspecifics. Based on the results of our habitat-preference analyses, we hypothesize that the dispersal of fungus-farming ants into dry or seasonally dry habitats may have been the key factor driving fungal isolation and subsequent coevolution. Reduced fungal outcrossing by ant dispersal into dry habitat is supported by the observations that leucocoprineaceous fungi, the group that includes most attine ant cultivar species, are most abundant and diverse in rainforests [40,41], and that all free-living conspecifics of attine cultivars have been collected only in the rainforests of Brazil and Panama [20,24]. Unable to escape the humid confines of their host nests [87,88], selection would eventually have driven higher attine fungi to become obligate ant nest specialists.

In addition to shifts into dry habitat, our results suggest fungus-farming ants expanded their range into Middle America and the Nearctic (figure 1) multiple times, with the earliest dispersal out of South America occurring around 27–22 Ma—well before the traditional estimate for the closure of the Isthmus of Panama (approx. 3 Ma) [71] (but see also [89–91]). Intriguingly, the sister group to the leaf-cutter ants is a lineage of completely Nearctic species in the *Trachymyrmex septentrionalis* group and the ancestor of the leaf-cutting ants probably originated in Middle America. If a shift into dry, inhospitable habitats provided the conditions for strict ant-fungus coevolution, it might also be the case that dispersal out of South America and into peripheral areas played a part as well. Movement into Middle America, and especially into the Nearctic, would have further isolated fungal cultivars from their parent populations and free-living progenitors. In the case of higher fungus farmers, geographical shifts would have the effect of isolating the ants from access to alternative species of higher attine fungi under cultivation by other higher attine species in the ancestral range.

Our results present a more detailed picture of the overall biogeographic history of fungus-farming ants. The tribe Attini, to which the fungus farmers belong, originated at about the same time as the K–Pg mass extinction event. Our phylogeny suggests that there was probably a burst of diversification at this time as evidenced by the short internodes subtending major clades and the fact that we could not confidently resolve relationships among most genus groups, even with genome-scale data. The fungus-farming ants originated shortly after the K–Pg extinction event between 61 Ma and 57 Ma, possibly during the post-extinction-event recovery period [92] and shortly before the early Eocene climatic optimum. It is during this time that the ants began their symbiosis with leucocoprineaceous fungi and lost their ability to synthesize arginine [27,33], committing them to agrarian life. Transitions from lower to other agricultural systems (five systems total; see figure 1) occurred after the terminal Eocene event (TEE) at approximately 35 Ma. This event involved a major drop in global temperature, which brought glaciers to Antarctica and began the expansion of drier habitats throughout the New World [93,94]. Although dry habitats such as grasslands have been present since the early Eocene [94], several studies have noted significant expansions of C4 grasses starting around 30 Ma [95–97], remarkably close in time to the origin of higher agriculture. It is also notable that we inferred a diversification rate increase in the lineage that includes all neoattines, minus a grade of several depauperate groups (*Cyatta, Kalathomyrmex*, *Mycetarotes* and *Mycetosoritis hartmanni*), at about the same time as the TEE. Given that most genus-level lineages and all derived agricultural systems originated after the TEE, it is plausible that this global cooling event spurred both attine ant lineage diversification and ant–fungus coevolution.

A critical, long-standing problem for understanding the evolution of ant agriculture has been identifying the non-fungus-growing sister lineage of the fungus-farming ants. The identity of the sister group is important because it could provide critical information about the behavioural, physiological or ecological precursors to fungus farming. Twelve different lineages have been variously proposed and, of these, morphological studies have supported the cryptic leaf-litter ant *Blepharidatta brasiliensis* as the sister group [98–100]. More recent molecular studies, however, have provided low to moderate support for a sister-group relationship between the fungus-farming ants and the subtribe Dacetina [23,32,42]. Using phylogenomic data and fundamentally different analytical paradigms, we found nearly unequivocal support for a sister-group relationship between the fungus-farming ants and the subtribe Dacetina.

The dacetines form a group of largely specialized predatory ants that occur in the Neotropical (*Acanthognathus, Daceton, Lenomyrmex*), Afrotropical (*Microdaceton*) and Australasian (*Colobostruma, Epopostrma* and *Mesostruma*) regions. Most of the genera have elongate, trap-jaw mandibles. Biological information for the dacetines is surprisingly limited, with the best-studied genera being *Acanthognathus* and *Daceton* [101–108]. Importantly, these ants have no known associations with fungi. The Dacetina and Attina do, however, share a few morphological and behavioural traits [104,109]. Most notably, both sister clades are highly specialized, the Dacetina specialized predators and the Attina specialized agriculturalists. Although these are behavioural extremes, it is possible they share a common historical origin. Given that both of these lineages evolved from generalized hunter–gatherers during the ‘nuclear winter’ that followed the K–Pg extinction event (figure 1) [92,110], it is possible that a reduction of generalized resources drove them to specialize, one on live prey and the other on fungi. As noted by Janzen [110], the best survivors of the K–Pg nuclear winter were probably those whose food did not directly depend on immediate photosynthesis. Specialization on fungi by the Attina may have been driven both by a reduction in generalized prey as well as by a proliferation of fungi [111].

## 5. Conclusion

The importance of animal and plant domestication to the rise of modern human civilization is well understood. Through a variety of mechanisms, including selective breeding and genetic isolation, humans increased food yield and nutritional value, fuelling the growth of civilizations. Similarly, in fungus-farming ants, the evolution of agriculture and subsequently of high-yield domesticated crops resulted in the rise of the ecologically dominant leaf-cutting ants, which form ‘superorganism’ colonies composed of millions of individuals. Unlike human agriculture, however, an explanation for how ants unconsciously domesticated their fungal cultivars has been highly uncertain. Our results provide the first evidence that fungal domestication occurred in dry habitats, suggesting an explanation for how fungal cultivars may have evolved into domesticated mutualists obligately dependent on their ant hosts.

## Supplementary Material

Appendix 1.

## Supplementary Material

Appendix 2.

## Supplementary Material

Appendix 3.

## Supplementary Material

Appendix 4.

## Supplementary Material

Appendix 5.

## Data accessibility

For all newly sequenced samples, raw sequence reads and Trinity contig assemblies representing UCE loci are available from the NCBI Sequence Read Archive and GenBank, respectively, under BioProject accession PRJNA379607. Additional data, including alignments, alignment supermatrices, tree files, partitioning files, BEAST xml files, trait and biogeography data files, and tables are available from Dryad (http://dx.doi.org/10.5061/dryad.d7pr6) [112].
