# Supplementary material for: Dry habitats were crucibles of domestication in the evolution of agriculture in ants
Source: Proc Biol Sci. 2017 Apr 12;284(1852):20170095. doi: 10.1098/rspb.2017.0095 (PMC5394666; doi:10.1098/rspb.2017.0095)
Supplement: Appendix 2. [file rspb20170095supp2.docx]

**Dry habitats were crucibles of domestication in the evolution of agriculture in ants**

**Appendix 2.** Supplementary methods, references, and tree figures.

Proceedings of the Royal Society B

**Author List:**

*Michael G. Branstetter^1,2^, Ana Ješovnik^2,3^, Jeffrey Sosa-Calvo^2,4¶^, Michael W. Lloyd^2^, Brant C. Faircloth^5^, Seán G. Brady^2^, *Ted R. Schultz^2^

^1^Department of Biology, University of Utah, Salt Lake City, UT 84112, USA

^2^Department of Entomology, National Museum of Natural History, Smithsonian Institution, Washington, D.C., 20560, USA

^3^Department of Entomology, University of Maryland, College Park, MD 20742, USA

^4^Center for Social Insect Research, School of Life Sciences, Arizona State University, Tempe, AZ 85287, USA

^5^Department of Biological Sciences and Museum of Natural Science, Louisiana State University, Baton Rouge, LA 70803, USA

***Corresponding Authors:**

Michael G. Branstetter
Email: mgbranstetter@gmail.com

Ted R. Schultz

Email: schultzt@si.edu

**Supplementary Methods**

***Library preparation, UCE enrichment, and sequencing***

To generate the UCE dataet we performed the following steps: DNA extraction, DNA shearing, library preparation, UCE enrichment, sample pooling, and sequencing. Most of the methods follow those presented in Faircloth et al. [1], and a detailed outline is presented here. We extracted DNA using Qiagen DNeasy Blood and Tissue kits (Qiagen Inc., Valencia, California, U.S.A.). However, for several rare samples that had been extracted prior to our study, we performed whole-genome amplification to increase available DNA using Illustra Ready-To-Go GenomiPhy HY (GE Healthcare). For all samples, we measured DNA concentration with a Qubit 2.0 fluorometer (Life Technologies Inc., Carlsbad, CA) and we input up to 500 ng of DNA into shearing and library preparation. We sheared the DNA to an average fragment distribution of 400-600 base pairs (bp) (verified on an agarose gel) using a Qsonica Q800R sonicator (Qsonica LLC, Newton, CT).

Following sonication, we constructed sequencing libraries using either the standard Library Preparation Kit or the newer Hyper Prep Kit from Kapa (Kapa Biosystems, Wilmington, MA). We performed all reactions at quarter volume except for the PCR step, which we assembled at full volume (50 μL). During library preparation we added custom, single-indexing Truseq-style barcode adapters to most samples [2]. However, for a few libraries, we used a custom, dual-indexing set of adapter-primers [3]. We assessed success of library preparation by measuring DNA concentration with Qubit and by visualizing the libraries on an agarose gel. For a subset of the samples, we removed adapter-dimers by performing a 0.7-0.8X bead cleaning using an AMPure substitute [4].

After library preparation, we pooled 6-10 libraries together at equimolar concentrations for enrichment. Pool concentration was adjusted to 147 ng/μl using a vacuum centrifuge and 3.4 μl of pool was input into the enrichment process. UCE enrichment was performed using a custom RNA bait library developed for use in Hymenoptera by Faircloth et al. [1] and synthesized by MYcroarray (MYcroarray, Ann Arbor, MI). The bait set includes 2,749 probes targeting 1,510 UCE loci. Enrichment followed a standardized, in-solution enrichment protocol (version 1.5; protocol available from <http://ultraconserved.org>). Enrichment incubation was performed at 65ºC for 24 hours.

Following enrichment, we measured the DNA concentration of pools using qPCR (Kapa qPCR reagents; ViiA7 instrument, Thermo Fisher Scientific Inc.) and we combined pools at equimolar ratios. No more than 110 individual samples were pooled together into the same sequencing pool. To remove fragments that were either too large or too small for sequencing, we size selected the pools to a range of 300-800 bp using a Blue Pippin size selection instrument (Sage Science, Beverly, MA). We sent size-selected pools to either the UCLA Neuroscience Genomics Core or Cornell’s Institute of Biotechnology for sequencing as single lanes on an Illumina HiSeq 2500 (2x150 rapid run; Illumina Inc., San Diego, CA).

***Bioinformatics and matrix preparation***

The sequencing centers demultiplexed and converted the raw illumina data from BCL to FASTQ format. Starting with the FASTQ files, we performed all initial bioinformatics steps, including read cleaning, assembly, and alignment, using the Phyluce v1.4 software package). We cleaned and trimmed raw reads using Illumiprocessor [5], which is a wrapper around Trimmomatic [6], and we assembled cleaned reads *de novo* using a Phyluce wrapper script (assemblo_trinity.py) around Trinity [7]. We selected Trinity for read assembly, because Faircloth et al. [1] demonstrated that it generates better assembles with UCE data than other popular assembly programs. After assembly, we mapped Trinity contigs to UCE loci (match_contigs_to_probes.py), with the “min-coverage” and “min-identity” options set to 50 and 70, respectively. These less stringent settings increased the average number of captured UCE loci by several hundred as compared to the default settings in Phyluce. Next, we used two scripts (get_match_counts.py and get_fastas_from_match_counts.py) to create a fasta file containing all taxa and UCE loci. During these steps we used the “incomplete-matrix” flag to allow matrices to have missing data, and we added UCE data from two genome-enabled taxa (*Atta cephalotes* and *Acromyrmex echinatior*). We aligned all loci individually using a wrapper script (seqcap_align_2.py) around the program MAFFT v7.130b (Katoh et al 2002). We used the “no-trim” and “incomplete-matrix” options during alignment to prevent the data from being trimmed and to include loci with missing data. After alignment, we used a script (get_gblocks_trimmed_alignments_from_untrimmed.py) to run Gblocks [8,9] on all aligned loci. We reduced the stringency of the default settings in Gblocks by adjusting the b1, b2, b3, and b4 options to 0.5, 0.5, 12, and 7, respectively. These settings were manually optimized by trial and error and visual inspection of alignments.

To determine an acceptable amount of missing data to include, we performed several rounds of matrix filtering followed by phylogenetic inference and assessment. To do this we used a script (get_only_loci_with_min_taxa.py) that filters loci for varying amounts of taxon occupancy (% samples required to be present in any given locus). We used the script to filter all loci for 100, 99, 95, 90, 75, 50, and 25% taxon occupancy. We then generated alignment stats using several scripts (get_align_summary_data.py and get_informative_sites.py) and generated a concatenated matrix for all levels of taxon occupancy (format_nexus_files_for_raxml.py). With the exception of the 100 and 99% filtered matrices (these included very few loci) we analyzed all concatenated matrices using RAxML v8.0.2 [10]. For each run we performed a rapid bootstrap analysis (N = 100) plus best tree search using GTR+Γ as the model of sequence evolution. We determined the “best” filtering level subjectively by considering matrix completeness, topology, and bootstrap scores. Following evaluation, we selected the 75% filtered alignment set as the primary alignment set for further analysis (“Attine-118T-F75”). The Attine-118T-F75 alignment set includes 950 loci and 652,774 bp of sequence data, of which 305,858 are parsimony informative.

***Phylogenetic analyses***

Using the Attine-118T-F75 alignment set, we investigated the effects of inference method and data partitioning on results. We excluded one taxon from this focal alignment set (*Paramycetophylax bruchi*), because it received few captured UCE loci (table S2, appendix A1). We did, however, include this taxon in a separate, 119-taxon analysis, described below.

For phylogeny estimation we compared maximum likelihood (ML), Bayesian inference (BI), and species tree (ST) approaches. For concatenated ML analyses we performed four analyses using RAxML v8 [10]: unpartitioned, partitioned by locus, partitioned by PartitionFinder v2.0 using the rcluster algorithm (PF; data pre-partitioned by locus ) [11,12], and partitioned by PF v2.0 using the kmeans algorithm [13]. For each analysis we executed a rapid bootstrap plus best tree search (“-f a” option) and we used the GTR+Γ model of sequence evolution (for both best tree and bootstrap searches). We performed 100 bootstrap replicates for all searches plus we performed an additional search on the kmeans-partitioned matrix for 500 replicates. To check that RAxML found the best tree topology, we performed 20 additional best tree searches on the kmeans-partitioned matrix using random starting trees (“-d” option). To address the concern that bootstrap scores can be misleading with phylogenomic data [14], we performed a jackknifing analysis, in which we randomly sampled subsets of genes multiple times. We used a Phyluce script (randomly_sample_and_concatenate.py) to randomly sample 100 UCE loci from the Attine-118T-F75 alignment set, 100 times. We concatenated each set of 100 random loci and inferred the best ML tree using RAxML (“-f d” option, unpartitioned, GTR+Γ). We then concatenated the resulting best trees into a single file and generated an extended majority rule consensus tree with support values (“-J MRE” option).

For Bayesian inference we used ExaBayes v1.4.1 [15] and performed unpartitioned and partitioned searches on the concatenated data matrix. For all searches we executed four independent runs, each with four coupled chains (one cold and three heated chains). We ran the unpartitioned searches for a total of 500,000 generations and left all other parameters at default values. For the partitioned searches we used the same kmeans-partitioning scheme that we used with RAxML. We selected kmeans because the tree resulting from the kmeans-partitioned ML analysis had the highest likelihood and reasonable branch length estimates (table S5, appendix A1). For the partitioned BI searches, we performed two separate analyses, one with parsimony starting trees and one with random starting trees. For both analyses we linked branch lengths across partitions and allowed the runs to progress for 4 million generations. We assessed burn-in, convergence among runs, and run performance by examining log files with the Tracer v1.6.0 [16]. We computed consensus trees using the “consense” utility, which is included in the ExaBayes package.

To examine the possible influence of gene tree discordance on our results we performed gene-tree-species-tree estimation using the program ASTRAL v4.8.0 [17,18]. This program is particularly suitable to our data, because it can handle large, genome-scale datasets and allows for missing data. To begin, we ran PartitionFinder 2.0 on each UCE locus using the kmeans algorithm. We then used RAxML to generate partitioned gene trees with bootstrap support (best tree plus rapid bootstrap search, 200 replicates). We calculated mean bootstrap support for each gene tree using a R script modified from [19], and we used only the 500 best gene trees (highest mean bootstrap scores) as input into ASTRAL. This was done to reduce any noise that might be present in the species tree analysis from uninformative loci, a problem that has been demonstrated in other data sets [20]. We ran the ASTRAL analysis with 200 multi-locus bootstrap replicates [21].

To explore our data for other potential biases, we generated two additional matrices using the Attine-118T-F75 alignment set. First, we used the program BaCoCa [22] to identify any loci exhibiting significant deviations from base composition heterogeneity (chi-squared test, p < 0.05). After removing biased loci (36 total), we concatenated the remaining 914 loci for analysis (“Attine-118T-F75-975”). Second, to control for either base composition heterogeneity or saturation, we used a Phyluce script to convert the concatenated Attine-118T-F75 matrix to RY-coding (“Attine-118T-F75-RY”). We analyzed both of the above matrices with RAxML (unpartitioned, best tree plus rapid bootstrap search, GTR+Γ, 100 bootstrap replicates).

To place *Paramycetophylax bruchi* (excluded due to poor UCE capture) in the attine tree we performed one additional analysis with this taxon included. We generated a new set of alignments filtered at 75% taxon occupancy (“Attine-119T-F75”) and a concatenated matrix and performed an unpartitioned analysis in RAxML (best tree plus rapid bootstrap search, GTR+Γ, 100 bootstrap replicates).

***Divergence dating***

We used BEAST v1.8.2 (Drummond et al. 2012) to generate a time tree for the evolution of fungus-farming ants. To calibrate the analysis, we used nine fossil calibrations, and one secondary calibration (table S6, appendix A1). Due to computational challenges arising from having both lots of taxa and lots of sequence data, we developed a two-part strategy to decrease computation time: (1) we used a constraint tree and turned off tree search operators and (2) we used subsets of UCE loci rather than the entire matrix. For the constraint tree, we used the topology that was inferred to be the best in all of the partitioned RAxML analyses (by locus, rcluster, and kmeans). To generate the data subsets, we used a Phyluce script (randomly_sample_and_concatenate.py) to randomly sample 20 UCE loci five times. For each of the five matrices, we performed two independent BEAST runs, each progressing for 50 million generations, sampling every one thousand generations. In addition, we performed one run in which the data were removed so that the MCMC search sampled from the prior distribution only. All runs were done on unpartitioned concatenated matrices. For the clock model we selected uncorrelated lognormal, for the substitution model we used GTR+Γ, and for the tree prior we used a birth-death model. For the ucld.mean prior we used an exponential distribution with mean 10.0. All other priors were left at default values. We assessed burn-in, convergence among runs, and run performance by examining log files with Tracer v1.6. After removing burn-in, we combined trees using LogCombiner, and generated a maximum clade credibility tree using TreeAnnotator (both programs included in BEAST package). To evaluate our approach of analyzing multiple data subsets, we generated chronograms for each of the five matrices separately and for all runs combined, and we compared dates across runs and with other recent molecular studies on fungus-farming ants.

***Historical biogeography***

We inferred the biogeographic history of the fungus-farming ants using the R package BioGeoBEARS (BGB) [23,24]. For the tree, we used the BEAST time tree pruned to include only the fungus-farming ants and their sister group. We coded taxa for the following areas: (A) Nearctic; (B) Middle America (including the Caribbean); (C) South America; (D) Afrotropics; and (E) Australasia. Using BGB we compared six different biogeographic models: DEC [25], DEC+J, DIVALIKE [26], DIVALIKE+J, BAYAREA [27], and BAYAREALIKE+J. For each model we performed a time-stratified analysis using the time periods (0-5 Ma, 5-35 Ma, 35-65 Ma), which correspond to post-closure of the Isthmus of Panama [28], pre-closure of the Isthmus of Panama, and pre-glaciation of Antarctica [29], respectively. For all time periods we set the probability of dispersing between adjacent, connected areas as 1.0 and between non-adjacent areas as 0.001. For dispersal between areas B and C we assigned probabilities of 1, 0.5, and 0.1 for the three time periods. For dispersal between C and D, C and E, and D and E we assigned probabilities of 0.001, 0.001, and 0.1 for the same time periods. We disallowed non-adjacent areas as possibilities in the analysis (AC, AD, AE, BD, BE, CD, CE, DE), except for CD, CE, and DE, which we allowed only during the 35-65 Ma time period. We set the maximum number of areas a species could occupy to two areas. We chose the best-fitting model by comparing model likelihoods and by performing likelihood-ratio tests.

***Diversification rate analyses***

We investigated diversification dynamics in the fungus-farming ants using two approaches. We tested for significant shifts in diversification rates across the entire tree using the R package TreePar [30]. TreePar is a ML-based program that allows for non-constant diversification rates and incomplete taxon sampling. It fits a birth-death model to dated trees and allows rates to change as a function of time. We ran the program using the “bd.shifts.optim” function and used the dated consensus tree from BEAST, with all outgroups pruned, as the input tree. We set extant diversity at 21% to account for incomplete species sampling (77 of 373 species); includes an estimate of undescribed species) and we set the program to test for a maximum of five rate shifts at 0.1 My intervals ranging from zero to 57 Ma. We selected the best-fitting model by performing likelihood-ratio tests with significance assessed by p-values below 0.05.

After assessing tree-wide rate shifts, we tested for rate shifts among lineages using the Bayesian program BAMM v2.5 [31–34] (see also [35,36]) and the accompanying R package BAMMtools [37]. BAMM is capable of modeling complex diversification dynamics by exploring many models simultaneously using reversible jump Markov Chain Monte Carlo (rjMCMC). A major advantage over likelihood-based approaches is that this method allows one to assess support for different rate-shift configurations using posterior probabilities and Bayes Factors. It also allows for incomplete taxon sampling in a way that does not require collapsing or pruning branches in the input tree.

For input into BAMM we used the BEAST consensus chronogram with all outgroups pruned. To account for non-random, incomplete taxon sampling we input a sampling probability file (table S7, appendix A1), in which we assigned all terminals to clades roughly corresponding to genera and we gave each clade a sampling probability based on the number of species sampled and the number of extant species in that clade (described and undescribed). To select appropriate priors for the BAMM analysis, we used the “setBAMMpriors” function in BAMMtools on the input time tree. Using the prior settings, we then ran the BAMM analysis with four chains for 200,000,000 generations, sampling event data every 10,000 generations. To improve MCMC sampling of the parameter space we adjusted the poisson rate prior to 0.5. After the analysis, we assessed burnin, run convergence, and ESS values for all parameters using the R package CODA [38]. We then explored the post-burnin output using BAMMtools and selected the best rate-shift configuration by assessing posterior probabilities and Bayes Factors.

**Supplementary References**

1. Faircloth BC, Branstetter MG, White ND, Brady SG. 2015 Target enrichment of ultraconserved elements from arthropods provides a genomic perspective on relationships among Hymenoptera. *Mol. Ecol. Resour.* **15**, 489–501. (doi:10.1111/1755-0998.12328)

2. Faircloth BC, Glenn TC. 2012 Not all sequence tags are created equal: designing and validating sequence identification tags robust to indels. *PLoS One* **7**, e42543. (doi:10.1371/journal.pone.0042543)

3. Glenn TC et al. 2016 Adapterama I: Universal stubs and primers for thousands of dual-indexed Illumina libraries (iTru & iNext). *bioRxiv*. 1–33. (doi:http://dx.doi.org/10.1101/049114)

4. Rohland N, Reich D. 2012 Cost-effective, high-throughput DNA sequencing libraries for multiplexed target capture. *Genome Res.* **22**, 939–946. (doi:10.1101/gr.128124.111)

5. Faircloth BC. 2013 illumiprocessor: a trimmomatic wrapper for parallel adapter and quality trimming. Available from http://dx.doi.org/10.6079/J9ILL

6. Bolger AM, Lohse M, Usadel B. 2014 Trimmomatic: a flexible trimmer for Illumina sequence data. *Bioinformatics* **30**, 2114–2120. (doi:10.1093/bioinformatics/btu170)

7. Grabherr MG et al. 2011 Full-length transcriptome assembly from RNA-Seq data without a reference genome. *Nat. Biotechnol.* **29**, 644–52. (doi:10.1038/nbt.1883)

8. Castresana J. 2000 Selection of conserved blocks from multiple alignments for their use in phylogenetic analysis. *Mol. Biol. Evol.* **17**, 540–552. (doi:10.1093/oxfordjournals.molbev.a026334)

9. Talavera G, Castresana J. 2007 Improvement of phylogenies after removing divergent and ambiguously aligned blocks from protein sequence alignments. *Syst. Biol.* **56**, 564–577. (doi:10.1080/10635150701472164)

10. Stamatakis A. 2014 RAxML version 8: A tool for phylogenetic analysis and post-analysis of large phylogenies. *Bioinformatics* **30**, 1312–1313. (doi:10.1093/bioinformatics/btu033)

11. Lanfear R, Calcott B, Ho SYW, Guindon S. 2012 PartitionFinder: Combined selection of partitioning schemes and substitution models for phylogenetic analyses. *Mol. Biol. Evol.* **29**, 1695–1701. (doi:10.1093/molbev/mss020)

12. Frandsen PB, Calcott B, Mayer C, Lanfear R. 2015 Automatic selection of partitioning schemes for phylogenetic analyses using iterative k-means clustering of site rates. *BMC Evol. Biol.* **15**, 13. (doi:10.1186/s12862-015-0283-7)

13. Lanfear R, Frandsen PB, Wright AM, Senfeld T, Calcott B. 2016 PartitionFinder 2: New methods for selecting partitioned models of evolution for molecular and morphological phylogenetic analyses. *Mol. Biol. Evol.* **34**, 772-773. (doi:10.1093/molbev/msw260)

14. Salichos L, Rokas A. 2013 Inferring ancient divergences requires genes with strong phylogenetic signals. *Nature* **497**, 327–331. (doi:10.1038/nature12130)

15. Aberer AJ, Kobert K, Stamatakis A. 2014 ExaBayes: massively parallel Bayesian tree inference for the whole-genome era. *Mol. Biol. Evol.* **31**, 2553–2556. (doi:10.1093/molbev/msu236)

16. Rambaut A, Suchard MA, Xie D, Drummond AJ. 2014 Tracer v1.6, Available from http://beast.bio.ed.ac.uk/Tracer.

17. Mirarab S, Reaz R, Bayzid MS, Zimmermann T, Swenson MS, Warnow T. 2014 ASTRAL: genome-scale coalescent-based species tree estimation. *Bioinformatics* **30**, i541–i548. (doi:10.1093/bioinformatics/btu462)

18. Mirarab S, Warnow T. 2015 ASTRAL-II: Coalescent-based species tree estimation with many hundreds of taxa and thousands of genes. *Bioinformatics* **31**, i44–i52. (doi:10.1093/bioinformatics/btv234)

19. Borowiec ML, Lee EK, Chiu JC, Plachetzki DC. 2015 Extracting phylogenetic signal and accounting for bias in whole-genome data sets supports the Ctenophora as sister to remaining Metazoa. *BMC Genomics* **16**, 987. (doi:10.1186/s12864-015-2146-4)

20. Meiklejohn KA, Faircloth BC, Glenn TC, Kimball RT, Braun EL. 2016 Analysis of a rapid evolutionary radiation using ultraconserved elements (UCEs): Evidence for a bias in some multispecies coalescent methods. *Syst. Biol.* **65**, 612–627. (doi:10.1093/sysbio/syw014)

21. Seo TK. 2008 Calculating bootstrap probabilities of phylogeny using multilocus sequence data. *Mol. Biol. Evol.* **25**, 960–971. (doi:10.1093/molbev/msn043)

22. Kück P, Struck TH. 2014 BaCoCa - A heuristic software tool for the parallel assessment of sequence biases in hundreds of gene and taxon partitions. *Mol. Phylogenet. Evol.* **70**, 94–98. (doi:10.1016/j.ympev.2013.09.011)

23. Matzke NJ. 2013 BioGeoBEARS: Biogeography with Bayesian (and likelihood) evolutionary analysis in R scripts. University of California, Berkeley, Berkeley, CA.

24. Matzke NJ. 2014 Model selection in historical biogeography reveals that founder-event speciation is a crucial process in island clades. *Syst. Biol.* **63**, 951–970. (doi:10.1093/sysbio/syu056)

25. Ree RH, Smith SA. 2008 Maximum likelihood inference of geographic range evolution by dispersal, local extinction, and cladogenesis. *Syst. Biol.* **57**, 4–14. (doi:10.1080/10635150701883881)

26. Sanmartín I, Enghoff H, Ronquist F. 2001 Patterns of animal dispersal, vicariance and diversification in the Holarctic. *Biol. J. Linn. Soc.* **73**, 345–390. (doi:10.1006/bijl.2001.0542)

27. Landis MJ, Matzke NJ, Moore BR, Huelsenbeck JP. 2013 Bayesian analysis of biogeography when the number of areas is large. *Syst. Biol.* **62**, 789–804. (doi:10.1093/sysbio/syt040)

28. O’Dea A et al. 2016 Formation of the Isthmus of Panama. *Sci. Adv.* **2**, 1–12. (doi:10.1126/sciadv.1600883)

29. Zachos J, Pagani M, Sloan L, Thomas E, Billups K. 2001 Trends, rhythms, and aberrations in global climate 65 Ma to present. *Science.* **292**, 686–693.

30. Stadler T. 2011 Mammalian phylogeny reveals recent diversification rate shifts. *Proc. Natl. Acad. Sci. USA* **108**, 6187–6192. (doi:10.1073/pnas.1016876108)

31. Rabosky DL. 2014 Automatic detection of key innovations, rate shifts, and diversity-dependence on phylogenetic trees. *PLoS One* **9**, e89543. (doi:10.1371/journal.pone.0089543)

32. Rabosky DL, Santini F, Eastman J, Smith SA, Sidlauskas B, Chang J, Alfaro ME. 2013 Rates of speciation and morphological evolution are correlated across the largest vertebrate radiation. *Nat. Commun.* **4**, 1958. (doi:10.1038/ncomms2958)

33. Rabosky DL, Donnellan SC, Grundler M, Lovette IJ. 2014 Analysis and visualization of complex macroevolutionary dynamics: an example from Australian scincid lizards. *Syst. Biol.* **63**, 610–627. (doi:10.1093/sysbio/syu025)

34. Shi JJ, Rabosky DL. 2015 Speciation dynamics during the global radiation of extant bats. *Evolution.* **69**, 1528–1545. (doi:10.1111/evo.12681)

35. Moore BR, Höhna S, May MR, Rannala B, Huelsenbeck JP. 2016 Critically evaluating the theory and performance of Bayesian analysis of macroevolutionary mixtures. *Proc. Natl. Acad. Sci. USA* **113**, 9569–9574. (doi:10.1073/pnas.1518659113)

36. Rabosky DL, Mitchell JS, Chang J. 2017 Is BAMM flawed? Theoretical and practical concerns in the analysis of multi-rate diversification models. *Syst. Biol.* **syx037**. (doi:10.1093/sysbio/syx037)

37. Rabosky DL, Grundler M, Anderson C, Title P, Shi JJ, Brown JW, Huang H, Larson JG. 2014 BAMMtools : an R package for the analysis of evolutionary dynamics on phylogenetic trees. *Methods Ecol. Evol.* **5**, 701–707. (doi:10.1111/2041-210X.12199)

38. Plummer M, Best N, Cowles K, Vines K. 2006 CODA: Convergence Diagnosis and Output Analysis for MCMC. *R News* **6**, 7–11.

39. Nygaard S et al. 2011 The genome of the leaf-cutting ant *Acromyrmex* *echinatior* suggests key adaptations to advanced social life and fungus farming. *Genome Res.* **21**, 1339–1348. (doi:10.1101/gr.121392.111.10)

40. Suen G et al. 2011 The genome sequence of the leaf-cutter ant *Atta cephalotes* reveals insights into its obligate symbiotic lifestyle. *PLoS Genet.* **7**, e1002007. (doi:10.1371/journal.pgen.1002007)

41. Ješovnik A, Sosa-Calvo J, Lloyd MW, Branstetter MG, Schultz TR. 2017 Phylogenomic species delimitation and host-symbiont coevolution in the fungus-farming ant genus *Sericomyrmex* (Hymenoptera: Formicidae): Ultraconserved elements (UCEs) resolve a recent radiation. *Syst. Ent.* **Early View**. (doi:10.1111/syen.12228)

42. Ward PS, Brady SG, Fisher BL, Schultz TR. 2015 The evolution of myrmicine ants: phylogeny and biogeography of a hyperdiverse ant clade (Hymenoptera: Formicidae). *Syst. Entomol.* **40**, 61–81. (doi:10.1111/syen.12090)

43. Dlussky GM, Rasnitsyn AP. 2009 Ants (Insecta: Vespida: Formicidae) in the Upper Eocene amber of Central and Eastern Europe. *Paleontol. J.* **43**, 1024–1042. (doi:10.1134/S0031030109090056)

44. Wheeler WM. 1915 The ants of the Baltic Amber. *Schriften der Phys. Gesellschaft zu Königsb.* **55**, 1–142.

45. de Andrade ML, Baroni Urbani C. 1999 Diversity and adaptation in the ant genus *Cephalotes*, past and present. *Stuttg. Beitr. Naturkd. Ser. B (Geol. Paläontol.)* **271**, 1–889.

46. Carpenter FM. 1930 The fossil ants of North America. *Bull. Museum Comp. Zool.* **70**, 1–66.

47. Wilson EO. 1988 The biogeography of the West Indian ants (Hymenoptera: Formicidae). In *Zoogeography of Caribbean insects* (ed JK Liebherr), pp. 214–230. Ithaca, NY: Cornell University Press.

48. Baroni Urbani C, de Andrade ML. 1994 First description of fossil Dacetini ants with a critical analysis of the current classification of the tribe (Amber Collection Stuttgart: Hymenoptera, Formicidae. VI: Dacetini). *Stuttg. Beitr. Naturkd. Ser. B (Geol. Paläontol.)* **198**, 1–65.

49. Schultz TR. 2007 The fungus-growing ant genus *Apterostigma* in Dominican amber. *Mem. Am. Entomol. Inst.* **80**, 425–436.

50. Schultz TR, Brady SG. 2008 Major evolutionary transitions in ant agriculture. *Proc. Natl. Acad. Sci. USA* **105**, 5435–5440. (doi:10.1073/pnas.0711024105)

51. de Andrade ML. 2003 First descriptions of two new amber species of *Cyphomyrmex* from Mexico and the Dominican Republic (Hymenoptera: Formicidae). *Beitr. zur Entomol.* **53**, 131–139.

52. Baroni Urbani C. 1980 First description of fossil gardening ants (Amber collection Stuttgart and Natural History Museum Basel; Hymenoptera: Formicidae. I: Attini). *Stuttg. Beitr. Naturkd. Ser. B (Geol. Paläontol.)* **54**, 1–13.

**Supplementary Tree Figures**

**Figure S1.** ML tree from unpartitioned RAxML analysis of the Attine-118T-F75 data set.

**Figure S2.** ML tree from the by-locus partitioned RAxML analysis of the Attine-118T-F75 data set.

**Figure S3.** ML tree from the rcluster partitioned RAxML analysis of the Attine-118T-F75 data set.

**Figure S4.** ML tree from the kmeans partitioned RAxML analysis of the Attine-118T-F75 data set.

**Figure S5.** ML tree from unpartitioned RAxML analysis of the RY-coded Attine-118T-F75 data set.

**Figure S6.** Extended majority rule consensus tree generated from 100 ML topologies, each inferred in RAxML from 100 randomly selected and concatenated loci from the Attine-118T-F75 data set.

**Figure S7.** Bayesian tree from unpartitioned ExaBayes analysis of the Attine-118T-F75 data set.

**Figure S8.** Bayesian tree from kmeans partitioned ExaBayes analysis of the Attine-118T-F75 data set. Search started using random trees.

**Figure S9.** ML tree from unpartitioned RAxML analysis of the Attine-118T-F75-914 data set. For this data matrix, 36 loci were removed from the larger Attine-118T-F75 data set due to evidence of significant base composition heterogeneity among taxa.

**Figure S10.** Species tree from ASTRAL analysis of the 500 best UCE loci (best = highest mean bootstrap value. Support values form 200 multi-locus bootstrap replicates.

**Figure S11.** ML tree from unpartitioned RAxML analysis of the Attine-119T-F75 data set. The taxon *Paramycetophylax bruchi* (in red) was not included in other analyses because the number of recovered UCE loci for this taxon was much lower than the average number.


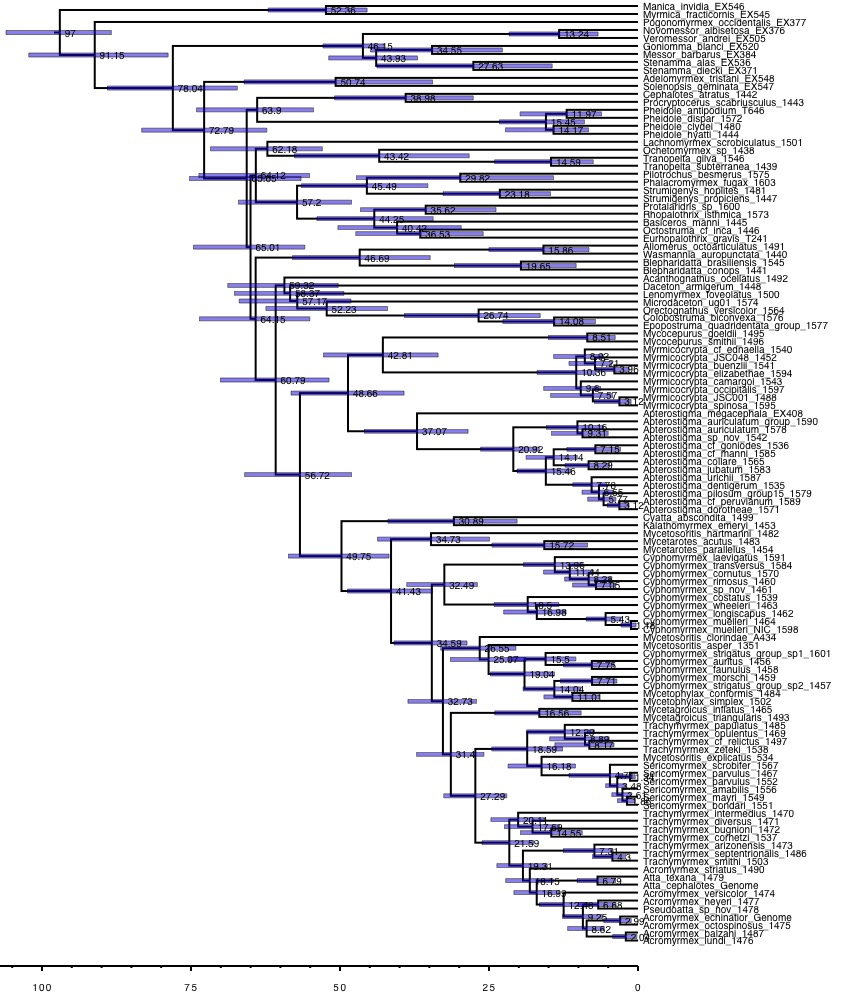


**Figure S12.** Time-calibrated tree from BEAST analysis using a constraint topology, five sets of 20 randomly selected loci, and nine fossil calibrations. Results from all analyses of the five different sets of loci were combined and averaged to generate this tree. Node labels are ages in millions of years ago and node bars indicate 95% highest posterior densities.
