# Supplementary material for: Dry habitats were crucibles of domestication in the evolution of agriculture in ants
Source: Proc Biol Sci. 2017 Apr 12;284(1852):20170095. doi: 10.1098/rspb.2017.0095 (PMC5394666; doi:10.1098/rspb.2017.0095)
Supplement: Appendix 3. [file rspb20170095supp3.docx]

**Dry habitats were crucibles of domestication in the evolution of agriculture in ants**

**Appendix 3.** Supplementary figures for reconstruction of ant agricultural systems.

Proceedings of the Royal Society B

**Author List:**

*Michael G. Branstetter^1,2^, Ana Ješovnik^2,3^, Jeffrey Sosa-Calvo^2,4¶^, Michael W. Lloyd^2^, Brant C. Faircloth^5^, Seán G. Brady^2^, *Ted R. Schultz^2^

^1^Department of Biology, University of Utah, Salt Lake City, UT 84112, USA

^2^Department of Entomology, National Museum of Natural History, Smithsonian Institution, Washington, D.C., 20560, USA

^3^Department of Entomology, University of Maryland, College Park, MD 20742, USA

^4^Center for Social Insect Research, School of Life Sciences, Arizona State University, Tempe, AZ 85287, USA

^5^Department of Biological Sciences and Museum of Natural Science, Louisiana State University, Baton Rouge, LA 70803, USA

***Corresponding Authors:**

Michael G. Branstetter
Email: mgbranstetter@gmail.com

Ted R. Schultz

Email: schultzt@si.edu

**Figure S12.** ML ancestral reconstruction of attine ant agricultural systems using the equal rates (ER) model.

**Figure S13.** ML ancestral reconstruction of attine ant agricultural systems using the symmetrical rates (SYM) model.

**Figure S14.** ML ancestral reconstruction of attine ant agricultural systems using the all rates different (ARD) model.
