# Supplementary material for: Dry habitats were crucibles of domestication in the evolution of agriculture in ants
Source: Proc Biol Sci. 2017 Apr 12;284(1852):20170095. doi: 10.1098/rspb.2017.0095 (PMC5394666; doi:10.1098/rspb.2017.0095)
Supplement: Appendix 4. [file rspb20170095supp4.docx]

**Dry habitats were crucibles of domestication in the evolution of agriculture in ants**

**Appendix 4.** Supplementary figures for all BioGeoBEARS analyses (biogeography and habitat).

Proceedings of the Royal Society B

**Author List:**

*Michael G. Branstetter^1,2^, Ana Ješovnik^2,3^, Jeffrey Sosa-Calvo^2,4¶^, Michael W. Lloyd^2^, Brant C. Faircloth^5^, Seán G. Brady^2^, *Ted R. Schultz^2^

^1^Department of Biology, University of Utah, Salt Lake City, UT 84112, USA

^2^Department of Entomology, National Museum of Natural History, Smithsonian Institution, Washington, D.C., 20560, USA

^3^Department of Entomology, University of Maryland, College Park, MD 20742, USA

^4^Center for Social Insect Research, School of Life Sciences, Arizona State University, Tempe, AZ 85287, USA

^5^Department of Biological Sciences and Museum of Natural Science, Louisiana State University, Baton Rouge, LA 70803, USA

***Corresponding Authors:**

Michael G. Branstetter
Email: mgbranstetter@gmail.com

Ted R. Schultz

Email: schultzt@si.edu

**Figure S15.** Ancestral areas estimated with BioGeoBEARS using the DEC model. Node labels indicate the most likely ancestral area.

**Figure S16.** Ancestral areas estimated with BioGeoBEARS using the DEC model. Pie charts on nodes indicate the relative probabilities of ancestral areas.

**Figure S17.** Ancestral areas estimated with BioGeoBEARS using the DEC+J model. Node labels indicate the most likely ancestral area.

**Figure S18.** Ancestral areas estimated with BioGeoBEARS using the DEC+J model. Pie charts on nodes indicate the relative probabilities of ancestral areas.

**Figure S19.** Ancestral areas estimated with BioGeoBEARS using the DIVALIKE model. Node labels indicate the most likely ancestral area.

**Figure S20.** Ancestral areas estimated with BioGeoBEARS using the DIVALIKE model. Pie charts on nodes indicate the relative probabilities of ancestral areas.

**Figure S21.** Ancestral areas estimated with BioGeoBEARS using the DIVALIKE+J model. Node labels indicate the most likely ancestral area.

**Figure S22.** Ancestral areas estimated with BioGeoBEARS using the DIVALIKE+J model. Pie charts on nodes indicate the relative probabilities of ancestral areas.

**Figure S23.** Ancestral areas estimated with BioGeoBEARS using the BAYAREALIKE model. Node labels indicate the most likely ancestral area.

**Figure S24.** Ancestral areas estimated with BioGeoBEARS using the BAYAREALIKE model. Pie charts on nodes indicate the relative probabilities of ancestral areas.

**Figure S25.** Ancestral areas estimated with BioGeoBEARS using the BAYAREALIKE+J model. Node labels indicate the most likely ancestral area.

**Figure S26.** Ancestral areas estimated with BioGeoBEARS using the BAYAREALIKE+J model. Pie charts on nodes indicate the relative probabilities of ancestral areas.

**Figure S27.** Ancestral habitats estimated with BioGeoBEARS using the DEC model. Node labels indicate the most likely ancestral habitat.

**Figure S28.** Ancestral habitats estimated with BioGeoBEARS using the DEC model. Pie charts on nodes indicate the relative probabilities of ancestral habitat estimates.

**Figure S29.** Ancestral habitats estimated with BioGeoBEARS using the DEC+J model. Node labels indicate the most likely ancestral habitat.

**Figure S30.** Ancestral habitats estimated with BioGeoBEARS using the DEC+J model. Pie charts on nodes indicate the relative probabilities of ancestral habitat estimates.

**Figure S31.** Ancestral habitats estimated with BioGeoBEARS using the DIVALIKE model. Node labels indicate the most likely ancestral habitat.

**Figure S32.** Ancestral habitats estimated with BioGeoBEARS using the DIVALIKE model. Pie charts on nodes indicate the relative probabilities of ancestral habitat estimates.

**Figure S33.** Ancestral habitats estimated with BioGeoBEARS using the DIVALIKE+J model. Node labels indicate the most likely ancestral habitat.

**Figure S34.** Ancestral habitats estimated with BioGeoBEARS using the DIVALIKE+J model. Pie charts on nodes indicate the relative probabilities of ancestral habitat estimates.

**Figure S35.** Ancestral habitats estimated with BioGeoBEARS using the BAYAREALIKE model. Node labels indicate the most likely ancestral habitat.

**Figure S36.** Ancestral habitats estimated with BioGeoBEARS using the BAYAREALIKE model. Pie charts on nodes indicate the relative probabilities of ancestral habitat estimates.

**Figure S37.** Ancestral habitats estimated with BioGeoBEARS using the BAYAREALIKE+J model. Node labels indicate the most likely ancestral habitat.

**Figure S38.** Ancestral habitats estimated with BioGeoBEARS using the BAYAREALIKE+J model. Pie charts on nodes indicate the relative probabilities of ancestral habitat estimates.
